# Relevance and Diversity of *Nitrospira* Populations in Biofilters of Brackish RAS

**DOI:** 10.1371/journal.pone.0064737

**Published:** 2013-05-21

**Authors:** Myriam Kruse, Sabine Keuter, Evert Bakker, Eva Spieck, Till Eggers, André Lipski

**Affiliations:** 1 Department of Food Microbiology and Hygiene, Institute of Nutrition and Food Science, University of Bonn, Bonn, Germany; 2 Department of Microbiology and Biotechnology, Biocenter Klein Flottbek, University of Hamburg, Hamburg, Germany; 3 Department of Microbiology, University of Osnabrück, Osnabrück, Germany; 4 Department of Ecology, University of Osnabrück, Osnabrück, Germany; National University of Singapore, Singapore

## Abstract

Lithoautotrophic nitrite-oxidizing bacterial populations from moving-bed biofilters of brackish recirculation aquaculture systems (RAS; shrimp and barramundi) were tested for their metabolic activity and phylogenetic diversity. Samples from the biofilters were labeled with ^13^C-bicarbonate and supplemented with nitrite at concentrations of 0.3, 3 and 10 mM, and incubated at 17 and 28°C, respectively. The biofilm material was analyzed by fatty acid methyl ester - stable isotope probing (FAME-SIP). High portions of up to 45% of *Nitrospira*-related labeled lipid markers were found confirming that *Nitrospira* is the major autotrophic nitrite oxidizer in these brackish systems with high nitrogen loads. Other nitrite-oxidizing bacteria such as *Nitrobacter* or *Nitrotoga* were functionally not relevant in the investigated biofilters. *Nitrospira*-related 16S rRNA gene sequences were obtained from the samples with 10 mM nitrite and analyzed by a cloning approach. Sequence studies revealed four different phylogenetic clusters within the marine sublineage IV of *Nitrospira*, though most sequences clustered with the type strain of *Nitrospira marina* and with a strain isolated from a marine RAS. Three lipids dominated the whole fatty acid profiles of nitrite-oxidizing marine and brackish enrichments of *Nitrospira* sublineage IV organisms. The membranes included two marker lipids (16∶1 *cis*7 and 16∶1 *cis*11) combined with the non-specific acid 16∶0 as major compounds and confirmed these marker lipids as characteristic for sublineage IV species. The predominant labeling of these characteristic fatty acids and the phylogenetic sequence analyses of the marine *Nitrospira* sublineage IV identified organisms of this sublineage as main autotrophic nitrite-oxidizers in the investigated brackish biofilter systems.

## Introduction

In biofilters of recirculation aquaculture systems (RAS), nitrite oxidation by lithoautotrophic bacteria (NOB) is the most important process to prevent the cultivated organisms from intoxication with nitrite [1]. Nitrite is formed by ammonia-oxidizing bacteria (AOB) and ammonia-oxidizing archaea (AOA) during the first process of aerobic nitrification. Together with heterotrophic bacteria, the nitrifying communities form biofilms on carrier elements in biofilter systems [2]–[3]. Although recirculation aquaculture systems are increasing worldwide [4], we still lack a detailed understanding of the process of bacterial nitrification, and especially nitrite oxidation, in marine RAS [5].

The guild of NOB is phylogenetically heterogeneous and comprises bacteria of at least five different genera: *Nitrobacter*, *Nitrospina*, *Nitrococcus*, *Nitrospira* and the recently discovered *Nitrotoga*. Co-existence of two [6]–[10], or three [11]–[12] genera of NOB in the same habitat has previously been reported. NOB grow slowly due to the low energy yield during oxidation of nitrite [13]. Thus the successful cultivation of NOB is a challenging and time consuming task. Consequently, NOB cultures have only rarely been established in the laboratory [14].

Hovanec *et al.* [15], and Juretschko *et al.* [16], were first to identify *Nitrospira* as the dominant NOB in freshwater aquaria and wastewater treatment plants. Later this genus was also detected in marine aquaculture and aquaria systems [2]–[3], [17]. However, only two studies so far have found *Nitrospira* as the dominant nitrite oxidizer in marine aquacultural biofilters [5], [18]. Here, we aimed to expand the metabolic activity data from Keuter *et al*. [5] of marine RAS by analyzes of the metabolic activity of NOB in brackish RAS.

Metabolic activity of nitrite-oxidizing populations can be assessed with DNA-based or chemotaxonomic methods. A DNA-based method of assimilation of labeled substrates performed by Radajewski *et al.* [19] provided the characterization of the ecology and function of methanol-utilizing organisms from forest soil. The incorporation of labeled substrates followed by gas chromatography and combustion isotope ratio spectrometry (GC-C-IRMS) was first used by Boschker *et al.* [20] for acetate assimilation of sulphate-reducing bacteria. More recently, quadrupole GC-MS (gas chromatography coupled with mass spectrometry) analyses of labeled lipids with ^13^C-acetate have been conducted [21] to study soil associated bacteria and fungi. The GC-MS analysis is generally less sensitive compared to GC-C-IRMS, but GC-MS systems are readily available to most laboratories [22]. A method based on quadrupole GC-MS for the labeling with ^13^C-bicarbonate of autotrophic populations in complex environments was described by Knief *et al.* [23]. Lipid analyses with fatty acid profiling in combination with stable-isotope probing (FAME-SIP) are very sensitive, making this a suitable technique for the quantification of autotrophic ^13^C-assimilation in nitrite supplemented biofilter material. For the analyses of the nitrite-oxidizing genera *Nitrobacter*, *Nitrococcus*, *Nitrospira*, *Candidatus* Nitrotoga and *Nitrospina* [14] by FAME-SIP, reference data sets for marker lipids are available [10]–[11], [24]–[25]. Especially the genus *Nitrospira* can be differentiated from the other genera of NOB based on characteristic lipid markers. These NOB are characterized by varying combinations of specific major compounds, which are the palmitoleic acid isomers *cis*-7 and *cis*-11 [24]. The different content of the characteristic lipids has been proved to be a good indicator for the presence of *Nitrospira* populations.

To determine the relevant NOB in two moving-bed biofilters of a brackish (17–21 psu (practical salinity units)) recirculation aquaculture system (shrimp and barramundi), we used FAME-SIP after incubation of the nitrifying community with different nitrite concentrations and temperatures. The chemotaxonomic analyses were supplemented by a cloning approach to get in-depth information about the diversity and the taxonomic positions of *Nitrospira* populations. Further, the fatty acid profiles of enrichment cultures obtained from the biofilter of the shrimp RAS and from marine sediments of the Laptev Sea were determined in order to expand the marker lipids data set of marine *Nitrospira* species.

## Methods

### Samples and enrichments

The samples were collected from two brackish recirculation aquaculture systems (RAS) in Strande (Germany (Marifarm; EAP AquaCulture AG)).

One system comprised shrimp (*Penaeus vannamei)* tanks with a total volume of 189 m^3^. The moving-bed biofilter of this system has a volume of 11.3 m^3^ and was started in early 2007 with approx. 3 m^3^ black and white high density polyethylene (HDPE) biocarriers (type HX09KL, Stöhr, Marktrodach, Germany) with a total surface area of 836 m^2^/m^3^ and a protected surface area of 494 m^2^/m^3^. The white biocarriers were made of new HDPE material, and the black were made of recycled HDPE material with the addition of 3% carbon black (Figure S1).

In the second, smaller experimental system, barramundi (*Lates calcarifer*) was reared in four tanks of a total volume of 10.8 m^3^. This system was started in 2006. Here, the biofilter comprised of 10 tanks containing 160 l water each, of which two were filled with 96 l black HX09KL (recycled material) and two with white HX09KL (new material) biocarriers.

Both systems ran at 26 to 29°C, the oxygen concentration was between 6.5 and 7 mg/l and pH was kept between 6.8 and 7. Make up water was taken from the Baltic Sea nearby, filtered and ozonized.

For the whole cell fatty acids profiles, enrichment cultures were started with biocarriers from the biofilter of the shrimp system as inoculum. The carriers were separated in white and black ones; cells were removed by shaking vigorously with glass beads (1.7–2 mm) and incubated for several months in marine mineral salt medium [26] with 70% brackish sea water at 28°C. Consumption of nitrite (1 mM) was regularly monitored with the Griess–Ilosvay spot test [27] and supplemented with NaNO_2_ (2.5 M stock solution) if necessary. In addition, the marine *Nitrospira*-enrichment culture, “S11”, which originated from marine sediments of the Laptev Sea in the permafrost region of Siberia [28], was incubated in marine mineral salt medium supplemented with 0.3 mM NaNO_2_ as described above. This culture was grown at 10°C and 17°C, respectively. Whole cell fatty acid profiles were analyzed from biomass from the two enrichments (see below).

For labeling experiments and subsequent cloning of *Nitrospira*-specific DNA fragments, biocarriers from the shrimp biofilter and from both biofilter components of the barramundi system (white and black biocarriers, respectively) were taken in October 2008. Nitrite and nitrate concentrations in the shrimp aquaculture system were 97 and 300 µM respectively, as determined by ion pair chromatography (Elite LaChrom System, Hitachi, Krefeld, Germany [29]). We measured the potential nitrite oxidation rate following the protocol by Spieck and Lipski [14]. The maximum nitrification potential, calculated as the average of two parallels, was 400 nmol NO_2_ *h^−1^ per biocarrier of the shrimp biofilter. In the barramundi system 1000 µM nitrate was measured, while nitrite could not be detected. The potential nitrite oxidation rates at sampling time were 1000 (white) and 1400 (black) nmol NO_2_ *h^−1^ per biocarrier.

### Lipid analyses

#### Whole cell fatty acid profiles

Cells were harvested by centrifugation (10,000× g; 20 min) when dense flocs had developed. Fatty acid methyl esters (FAMEs) were prepared according to Sasser [30]. The analyses of the FAMEs by GC-MS were performed as described previously [24].

#### Labeling experiments

In 500 ml gastight flasks filled with 100 ml marine mineral medium [26] prepared with 70% seawater (Baltic Sea; Fischland/Darß, Germany), 100 biocarriers from the two barramundi biofilters (black [Bb] or white [Bw] biocarriers) and the biofilter of the shrimp system (mixed black and white biocarriers [Sh]) were incubated at temperatures of 17°C and 28°C with 20 mM NaH^13^CO_3_ and NaNO_2_ concentrations of 0.3, 3 and 10 mM, resulting in a total of 18 different incubations. Nitrite consumption was tested with the Griess–Ilosvay spot test [27] twice a week and initial concentrations were readjusted if necessary. The oxygen content of the gas phase above the suspension was measured regularly by subjecting 10 µl of gas phase samples to GC-MS. The fatty acids from the sample biomass were extracted when one of samples from the same biofilter, incubated with the same temperature, contained less than 5% oxygen in the gas phase above the medium. Then all three flasks with the different nitrite concentrations (0.3 mM, 3 mM and 10 mM) were harvested. This resulted in incubation times of 41 up to 55 days (17°C) and 27 up to 41 days (28°C). We obtained biomass by shaking the HDPE carriers with glass beads (2 mm) in a horizontal shaker at 400 rpm overnight. The suspended biomass was concentrated by centrifugation (10,000× g; 20 min).

Lipids were extracted from biomass and converted to fatty acid methyl esters (FAMEs) as described by Knief *et al*. [23]. The resulting FAMEs were analyzed by GC-MS, and the degree of labeling was quantified using the SIM-mode (single ion monitoring) as described previously [23]. The natural concentration of ^13^C in biofilm samples was analyzed from samples without added NaH^13^CO_3_ and these values were subtracted from those of ^13^C-labeled samples.

Significance of labeled amounts of fatty acids was calculated based on a one-tailed Student's *t*-test. The ^13^C-content (L) was calculated for unlabeled references from fatty acids of original biofilm samples. Based on these data the *t*-test was used for the calculation of threshold values (T). Labeling was significant with amounts L>T (p<0.1). The threshold value was calculated for the fatty acids of the biofilm samples with percentage of 3.7% (p<0.1). Therefore, all fatty acids of the biofilters samples with degree of labeling ≥4% were deemed to be labeled.

### 16S rRNA gene analyses

#### Primer design

*Nitrospira*-specific primer NS1036R (5′-GCAGCACCTGAGCTCGCT-3′) was designed by using the PROBE-DESIGN Tool from ARB ([31]; version ssu_jan04.corr_opt.arb [http://www.arb-home.de]). Specificity of the primer was checked with the online databases RDP II with the Probe Match function (Ribosomal Database Project; [32]) and NCBI with the BLAST function (Basic local alignment search tool; [33]). Optimization of the amplification conditions of the new primer NS1036R was performed with DNA from cells of *Nitrospira marina* 295 by gradient PCR. Under these conditions amplicons were also obtained from *Nitrospira moscoviensis* M-1 and *Candidatus* Nitrospira defluvii. As negative controls reverence strains from several phyla were analyzed: *Pseudomonas spec*. FB1, FB19 and FB22 (acc. nos. AM933495.1; AM933511.1; AM933513.1) for *Proteobacteria*, *Subtercola spec.* FB10 (acc. no. AM940948.1) for *Actinobacteria* and *Mucilaginibacter* spec. FB 14.2 (acc. no. AM933506.1) for *Bacteroidetes*. The sequences exhibited 5 or 7 mismatches to the primer sequence of NS1036R.

#### Sequences of the *Nitrospira* community

DNA was extracted (PowerSoil™ DNA Isolation Kit; MoBio Laboratories, Carlsbad, CA) from the cell material shaken off the biocarriers which had been incubated with 10 mM nitrite. *Nitrospira*-specific 16S rRNA gene sequence fragments were obtained with the primer NS1036R, in combination with non-specific general bacterial primer Jur8F [16]. The PCR was performed with 30 cycles in two steps, the first step with 10 cycles at 55°C annealing temperature and the second step with 20 cycles at 45°C. Resulting fragments were cloned using the pGEM®-T vector system (Promega, USA). Clone sequences were analyzed with the online databases RDP II with the “seqmatch” function (Ribosomal Database Project; [32]) and NCBI with the “BLAST” function (Basic local alignment search tool; [33]). Furthermore, the sequences were checked for chimera and sequence anomalies using the Pintail program [34]. The sequences were named according to their origin (Bw: barramundi white biocarrier, Bb: barramundi black biocarrier, Sh: shrimp biocarrier) and the incubation temperature (17 or 28°C). Sequences derived from the gas tight flask incubations were labeled with the appendix OS (acc. nos. HE793388 – HE793423).

In addition to the incubation with NaH^13^CO_3_ in gas tight flask, a further set of incubations of the biocarrier samples was prepared in Erlenmeyer flasks with normal caps and without supplemented ^13^C. Biofilm from the carriers was harvested in a horizontal shaker at 400 rpm overnight. DNA was extracted from the 10 mM nitrite samples and the 16S rRNA genes were cloned and sequenced according to Keuter *et al.* [5]. The “Sh” DNA extracts were amplified with the primer pair Jur8F/1036R [16], and “Bb” and “Bw” DNA extracts were amplified with the primer pair Jur8F/Ntspa1158R [16], [35]. Sequences resulting from this cloning approach are labeled with the appendix HH (acc. nos. JQ900181-JQ900201; JX028301).

## Results

### Whole cell fatty acids of enrichments

The enrichment cultures inoculated with black and white biocarriers from the shrimp system (Sh) consisted of 19–26% of the fatty acid 16∶1 *cis*7 and 14–19% of the lipid 16∶1 *cis*11. The major fatty acid composition of the enrichment culture from the Laptev Sea (S11) was similar (16∶1 *cis*7: 32%; 16∶1 *cis*11: 40%–44%). Additionally, all enrichments showed the lipid 16∶0 as major compound with percentages from 14 up to 39% (Table 1). Data of two previously analyzed *Nitrospira* cultures, strains *Nitrospira marina* 295 [24] and Ecomares 2.1 [5], which contained high percentages of the lipid 16∶1 *cis*7 (30–41%) in combination with 16∶1 *cis*11 (16–31%), are also presented in Table 1.

**Table 1:** Whole fatty acid profiles of marine enrichments and *Nitrospira marina* 295 (fatty acids in %).

	*Nitrospira marina* 295^a^	Ecomares 2.1^b^		Sh (black carrier)	Sh (white carrier)	S11	
Cultivation temperature	28°C	17°C	28°C	28°C	28°C	10°C	17°C
12∶0	0.6	-	-	-	-		-
14∶0	1.4	1.0	-	1.9	3.3	1.0	0.8
15∶0 iso	0.8	-	-	7.3	6.1	-	-
15∶0 anteiso	-	-	-	2.4	4.1	-	1.3
15∶0	-	-	-	-	2.4	-	-
16∶0 iso	-	-	-	2.6	-	0.7	1.7
16∶1 *cis*7	30.4	40.7	37.1	25.8	19.0	32.4	31.8
16∶1 *cis*9	-	-	-	2.1	-	2.4	1.0
16∶1 *cis*10	-	1.9	2.8	-	-	-	1.1
16∶1 *cis*11	15.5	30.8	19.8	18.9	13.8	44.2	40.3
16∶0	36.5	24.4	35.3	29.9	39.3	14.0	15.7
16∶0 11methyl	0.8	-	2.3	-	-	-	-
17∶0 iso	0.8	-	-	2.3	-	-	0.3
18∶1 *cis*9	1.8	-	-	1.8	-	0.8	0.7
18∶1 *cis*11	0.6	-	-	2.6	5.4	3.1	1.3
18∶0	8.7	2.2	2.8	2.5	3.4	1.3	1.5
19∶0 cyclo 9–10	-	-	-	-	3.1	-	0.6

a data from Lipski *et al.* [24].

b data from Keuter *et al.* [5].

### Major fatty acids and labeling of the community

**Figure 1:**
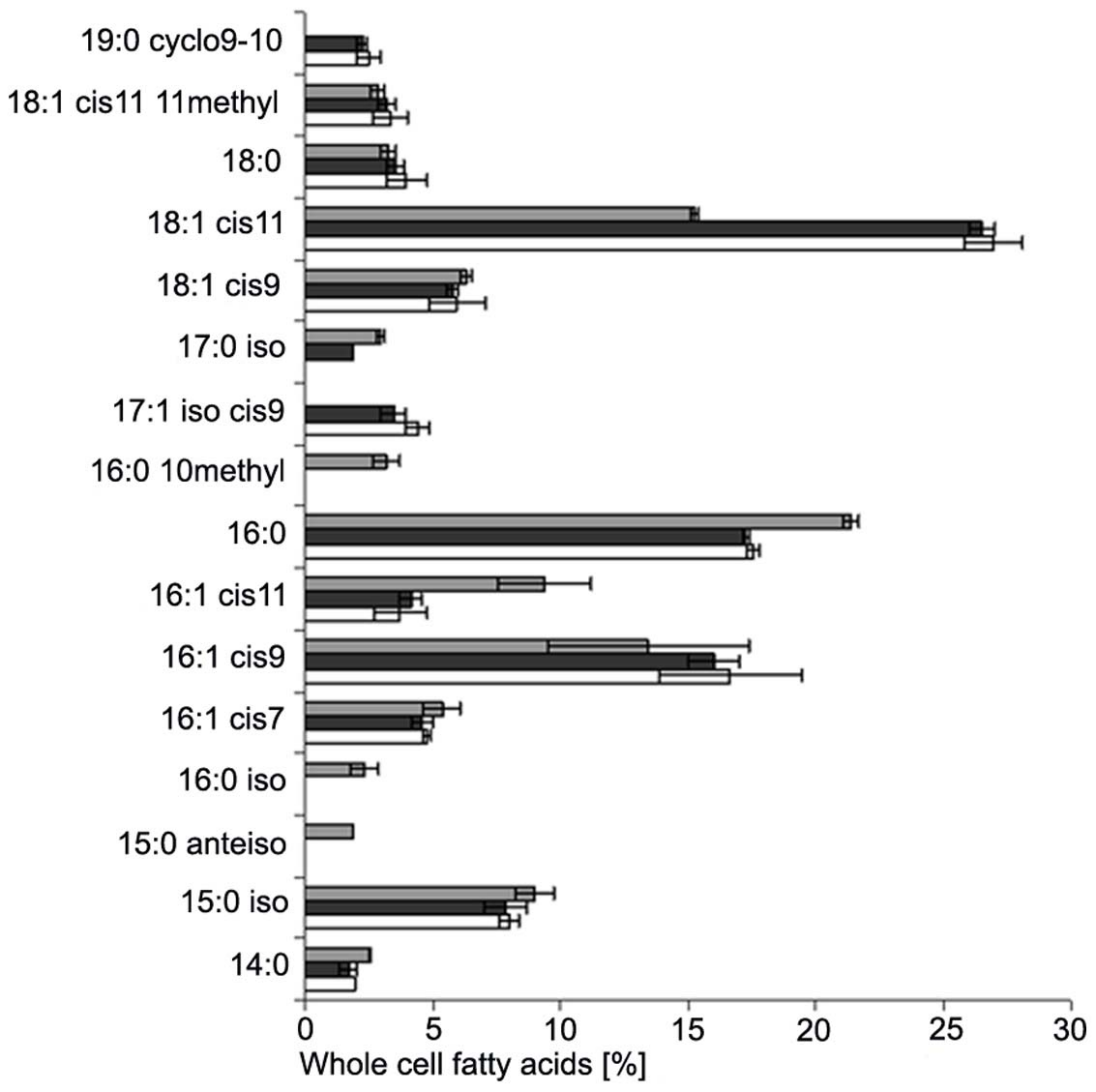
Whole fatty acid profile of the three original biofilter samples. Samples from the moving-bed biofilters were analyzed in parallels (error bars represent the standard deviation of n = 2 samples of each moving-bed system). Data from the barramundi biofilters are shown by white and black bars (white and black biocarriers), and those from the shrimp biofilter by grey bars.

The fatty acid profiles of the original moving-bed biofilter samples from the two barramundi biofilters and the shrimp biofilter were similar (for averages see Figure 1). The profiles were dominated by the fatty acids 16∶0, 16∶1 *cis*9 and 18∶1 *cis*11. Highest percentages of the lipid 18∶1 *cis*11 (up to 27%) were obtained from barramundi biofilter material and the highest percentage of the compound 16∶0 (21%) was obtained from shrimp biofilter material.

**Figure 2:**
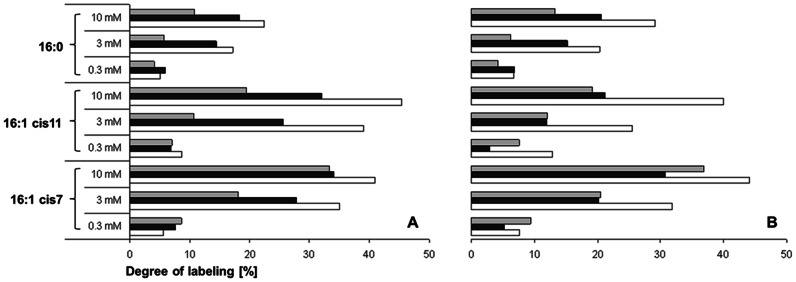
Degree of labeling of major compounds of the genus *Nitrospira*. Results from samples of the three moving-bed biofilters incubated at 17°C (A) and 28°C (B). Data from the barramundi biofilters are shown by white and black bars (white and black biocarriers), and those from the shrimp biofilter by grey bars.

Fatty acid profiles of the biomass in the three biofilter samples (Bb, Bw and Sh) were analyzed for the presence and isotopic content of selected diagnostic lipids after incubation with ^13^C-bicarbonate using different nitrite concentrations and temperatures. All major fatty acids of the known nitrite-oxidizing taxa were present in all these samples and showed significant portions of labeled fatty acids. The marker lipids of *Nitrospira*, fatty acid 16∶1 *cis*7, 16∶1 *cis*11 and 16∶0, showed highest ^13^C assimilation rates of up to 45% (p<0.005). In contrast, the dominating lipids of the genera *Nitrobacter* (18∶1 *cis*11), *Nitrococcus* (16∶1 *cis*9/18∶1 *cis*11), *Nitrospina* (14∶0/16∶1 *cis*9) and the taxon *Nitrotoga* (16∶1 *cis*9) showed only moderate incorporation of ^13^C in the course of the labeling experiments and never exceeded 13% (p<0.1) (Table 2). The degree of labeling of marker lipids for the genus *Nitrospira* increased with the nitrite concentration, i.e. the highest assimilation of ^13^C occurred in the samples with 10 mM nitrite at both temperatures (Figure 2). The samples from the barramundi filter with white biocarriers exhibited highest ^13^C assimilation rates (except for fatty acid 16∶0 and 16∶1 *cis*7 at 0.3 mM) (Figure 2) ranging between 32–44% for fatty acid 16∶1 *cis*7, 26–45% for fatty acid 16∶1 *cis*11 and 17–29% for fatty acid 16∶0.

**Table 2:** Labeled amounts of major compounds from nitrite-oxidizing bacteria.

	14∶0	16∶1 *cis*7	16∶1 *cis*9	16∶1 *cis*11	18∶1 *cis*11	16∶0
**Labeled amounts (%)** a	**6–13**	**30–44**	**6–13**	**19–45**	**6–10**	**11–29**
**Significance (** ***p*** **)**	<0.1	<0.005	<0.025	<0.005	<0.05	<0.01
**Dominating lipids**						
***Nitrospira*** ** IV** b		**++** c		**+(+)**		**+(+)**
***Nitrobacter***					**++**	**+**
***Nitrococcus***			**++**		**++**	**++**
***Nitrospina***	**++**		**++**			**+**
***Nitrotoga***			**++**			**++**

a range of the labeled amounts from the 3 moving bed biofilters (incubations with 10 mM nitrite and both used temperatures), see also Figure 2 for detailed information of *Nitrospira* marker lipids.

b marker lipids of *Nitrospira* sublineage IV, detailed information see Table 3.

c +: marker lipid with percentage ≤20%; ++: marker lipids with >20% of whole fatty acids for the respective taxon.

### Phylogeny of the *Nitrospira*-community

Phylogenetic analyses were performed on sequences from incubations with 10 mM nitrite in flasks without ^13^C (appendix HH) and gastight flasks supplemented with ^13^C-bicarbonat (appendix OS). The consumption rates of supplemented nitrite showed no relevant differences (data not shown) between the two types of incubation.

**Figure 3:**
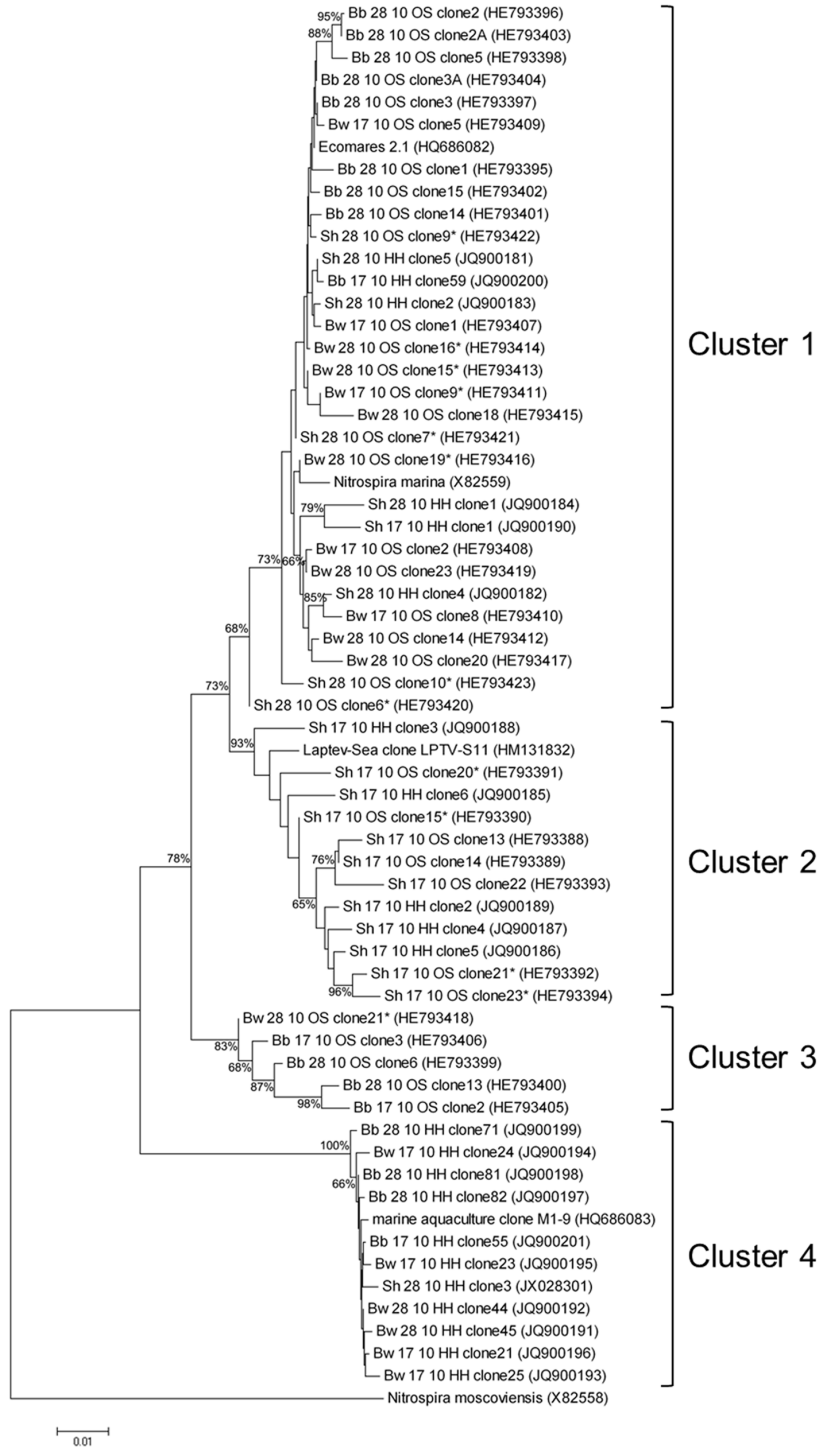
Phylogenetic tree based on 16S rRNA gene sequences. The tree was constructed by neighbor-joining algorithm showing four different clusters (1–4). Nodes ≥65% supported by bootstrap values (based on 1000 iterations). The tree shows sequences of the marine *Nitrospira* sublineage IV with clones of the brackish RAS biofilters. The sequence of *Nitrospira moscoviensis* from sublineage II was defined as outgroup. The sequences obtained from analyzed biofilters were named after their origin (Bw: barramundi white biocarrier; Bb: barramundi black biocarrier; Sh: shrimp biocarrier), the incubation temperature (17 or 28°C), as well as the appendix OS (acc. nos. HE793388 – HE793423) and HH (acc. nos. JQ900181-JQ900201; JX028301) for different cloning approaches. Sequences ≤800 bp was marked with “*”. Scale bar = 1% sequence divergence.

The cloning approach revealed four different clusters within sublineage IV of *Nitrospira* (Figure 3); cluster 1 comprised sequences of almost all analyzed biofilters affiliated with *Nitrospira marina* 295 (acc. no. X82559) and Ecomares 2.1, isolated from a marine RAS (acc. no. HQ686082) analyzed previously [5]. Cluster 2 included the sequence of the Laptev Sea sediment clone LPTV S11 (acc. no. HM131832), and comprised only cloned sequences of the Sh17 incubation, i.e. biocarriers from the shrimp biofilter with an incubation temperature of 17°C. Clusters 3 and 4 consisted of sequences from samples incubated in gas tight flasks (OS) or in open flasks (HH), respectively. Cluster 4 included a sequence of the enrichment culture M1–9 from a marine RAS (acc. no. HQ686083; [5]). Clusters 3 and 4 sequences differed from all sequences of cluster 1 and 2 in the nucleotides on position 482–487 (C–ACT in cluster 1 and 2, and T–GCC in cluster 3 and 4), according to the *E. coli* numbering [36].

## Discussion

### Metabolically active NOB in the biofilters

Members of the genus *Nitrospira* often co-exist with other NOB, or even dominate the nitrite oxidation in many engineered ecosystems such as wastewater treatment plants, aquaria and aquaculture biofilters, as indicated by several nucleic acid-based studies [2]–[3], [15]–[18], [37]. Fluorescence *in situ* hybridization (FISH) with rRNA-targeted probes is well practical for analyses of the microbial structure but is insufficient to determine the metabolic activity of cells. More detailed information on activity of a defined group of bacteria can be obtained by combining FISH with microradiography (MAR-FISH) [38]. However, in contrast to FAME-SIP the target sequences of autotrophic organisms must be known to obtain MAR-FISH signals from metabolic active groups. Because FAME-SIP is independent from available sequence information, it represents an appropriate method to analyze autotrophic communities in environmental samples. The results of the present study extended DNA-based findings for aerobic biofilters in brackish aquaculture biofilters of 17–21 psu salinity. Moreover, the labeling approach with FAME-SIP allows assessment of the metabolic activity of the whole autotrophic, nitrite-oxidizing community in the analyzed brackish aquaculture systems.

The straight chain fatty acids 16∶0, 16∶1 *cis*9 and 18∶1 *cis*11 continuously dominated the lipid profile, while other groups of fatty acids, polyunsaturated and iso-/anteiso-branched fatty acids, were detected in lower percentages for all samples. All these lipids occur mainly in heterotrophic cells. The lipid 18∶1 *cis*9, a compound of different heterotrophic cells, showed no significant assimilation of ^13^C in the course of the labeling experiments. The degree of labeling never exceeded 3.8% (data not shown). It is assumed that only active autotrophic cells, but not heterotrophic cells, show a considerable incorporation of label under the conditions we used [23].

The high degree of labeled *Nitrospira*-specific compounds, the *cis*-7 and *cis*-11 isomer of hexadecenoic acid, reflects the major role of *Nitrospira* cells in the biofilters. Marker lipids of the other nitrite-oxidizing autotrophic organisms, *Nitrobacter*, *Nitrospina*, *Nitrococcus* and *Candidatus* Nitrotoga were only moderately labeled (Table 2). The low degree of labeled *Nitrobacter* marker fatty acids at 10 mM nitrite contrasts the expectation that this NOB is often found as dominant NOB in habitats with high substrate availability [39]–[41], outcompeting *Nitrospira* which has high substrate affinity at low nitrite concentration [42]. However, *Nitrobacter*- and *Nitrotoga*-like bacteria were detected by specific PCR in samples from the shrimp (both) and the barramundi (only *Nitrotoga*) filters (data not shown), but no relevant activity could be assigned to these genera by the labeling approach.

In contrast to *Nitrobacter* (*Alphaproteobacteria*), *Nitrospira*-like bacteria of the deep-branching phylum *Nitrospirae* [43] grow slowly, and seem to be better adapted to low oxygen concentrations [44]. Their high nitrite affinities led to the assumption, that *Nitrospira* are K-strategists, which thrive at low nitrite concentrations [44]–[45]. The high metabolic activity of *Nitrospira* populations, especially at a concentration of 10 mM nitrite (Figure 2) is remarkable. In line with our finding, Maixner *et al.* [35] suggested a broader differentiation of ecotypes within the genus *Nitrospira* on an imaginary scale reaching from K-strategist to r-strategist. The *Nitrospira* in the studied brackish RAS hence might have been r-strategists, or/and bear currently unidentified features that are advantageous compared to other NOB.

*Nitrobacter* has rarely been reported to be a major NOB in marine habitats [8], [46]. Rather the marine genera *Nitrococcus* or *Nitrospina* would be theoretically more suitable as putative co-inhabitants or competitors in brackish or marine RAS. Both genera are tolerant to high nitrite concentrations [47] and *Nitrospina* is thought to be the most abundant nitrite oxidizer in the oceans [48], though sound data on the composition and distribution of NOB in the oceans are still lacking [49]. In contrast to the theory, no specific fatty acids of either of these NOB were labeled during our experiments and there was no hint for their relevance in the analyzed biofilters.

The similar pattern of labeled fatty acids at different nitrite concentrations suggests that only one dominant NOB was active under the different conditions. The amounts of the specific labeled acids of *Nitrospira* correlated positively with the nitrite concentrations. Both moving-bed-systems ran at 26 to 29°C, but the metabolic activity was not influenced by the incubation temperatures of 17 and 28°C. We detected similar activities at the original temperature of the system and the lower temperature conditions suggesting the autotrophic organisms are very resistant to a decrease in temperature. While the temperature difference of 9°C did not seem to have any influence on the activity of the NOB the type of the biocarriers did affect the activity of the NOB: In almost all incubations, the bacterial community on the white biocarriers of the barramundi biofilter incorporated more ^13^C-bicarbonate than the black carriers of the second barramundi biofilter, even though both biofilters were supplied by the same effluent water from the barramundi tanks. This is in accordance with results from a range of activity tests on black and white biocarriers from the shrimp biofilter between June 2008 and June 2009; the nitrite-oxidizing bacteria on the white biocarriers were more active than those on the black biocarriers (Figure S2). These results are interesting, since there is no difference in shape, structure or size of the white and black biocarriers. The only difference is that white biocarrier consisted of new HDPE material while the black HDPE biocarriers were made of recycled material pigmented with black carbon. Reasons for that are unclear and would require further studies.

### Phylogenetic relationship in sublineage IV

We assessed micro-diversity of *Nitrospira*-strains in the biofilters of the brackish RAS, by constructing a clone library using semi-specific *Nitrospira* primer pairs with DNA from the 10 mM nitrite incubations at both temperatures.

We assumed that the bacteria in the biofilters would be halotolerant or halophilic, since the systems were run with water from the Kiel Bight, a part of the Baltic Sea which can reach salinities of up to 30 psu. Our phylogenetic analyses showed that sequences from the analyzed biofilters belong to the marine *Nitrospira* sublineage IV, and some were highly similar to the few known marine cultures.

So far six sublineages of *Nitrospira*-strains are known, mostly containing only one described strain, each of which might be habitat specific [25], [37]. For instance, species of sublineage I (*Candidatus* N. defluvii-lineage), are found in wastewater treatment plants, while species of the sublineage VI (*N. calida*-lineage) seem to be restricted to hot springs. Sequences of sublineage IV have mainly been derived from marine habitats.

In various engineered ecosystems *Nitrospira*-related strains, from either different or the same sublineages, co-exist [18], [50]. Some authors associate population shifts or differences by physical or chemical factors like nitrite concentration, oxygen content or temperature [35], [51]–[52]. Brown *et al.* [53] discovered 16S rRNA gene sequences related to *Nitrospira marina*-like organisms and also sequences related to *Nitrospira moscoviensis*-like organisms in biofilters of a shrimp RAS. Quantification by qPCR showed higher abundances of *Nitrospira marina*-like organisms from sublineage IV than *Nitrospira moscoviensis*-like organisms from sublineage II. In the current study we found only sequences of *Nitrospira* from sublineage IV grouped into four different clusters using clone libraries of the barramundi and shrimp biocarriers.

Cluster 1 comprised the largest number of sequences originating from all incubations and included the strains *Nitrospira marina* and Ecomares 2.1. The latter was isolated from the moving-bed biofilter of a marine RAS in Büsum, Germany (North Sea water) [5]. However, a range of sequences confirmed three further clusters within the marine *Nitrospira* sublineage. Sequences from the colder incubation of the shrimp biocarriers were found clustering together with culture S11 (cluster 2). The culture S11 was observed to convert moderate nitrite concentrations (34–36 µM/day) irrespectively of the temperature (range between: 10–28°C) [28]. Population shift experiments with varying nitrite concentrations [35] revealed shifts within a few days, which is a relatively short time for nitrifiers. Therefore, the incubation at 17°C for approx. 55 days might have favored growth of bacteria of cluster 2.

Except for the colder incubation of biocarriers from the shrimps filter, cluster 4 comprised sequences from all incubation variants together with the sequence of enrichment culture M1-marine, a *Nitrospira* coexisting with the Ecomares 2.1 in the marine RAS run with North Sea water [5]. No representative of cluster III has been cultivated so far.

The genotypic information of marine and brackish habitats revealed high similarities of the sequences within the marine *Nitrospira* sublineage. This indicates that there are indeed different ecotypes in *Nitrospira* sublineage IV, although no grouping into marine and brackish strains could be observed. The marine sublineage IV is the only sublineage of *Nitrospira* with several isolated or highly enriched and physiologically studied representatives. Experiments on the strains S11 [28], Ecomares 2.1 [5], and *N. marina* 295 [26] revealed that members of this sublineage, even very close relatives such as *N. marina* 295 and Ecomares 2.1, can differ immensely in their substrate tolerances, temperature optima or substrate conversion rates. Such physiological diversity, a prerequisite for niche differentiation, might therefore explain the high phylogenetic micro-diversity. Micro-scale conditions in biofilms of biofilter systems can vary extremely over time and/or space [35], [44], enabling the co-existence of strains with differing physiological preferences for resources.

### New data sets of fatty acid profiles in sublineage IV

*Nitrospira*-typical fatty acids are used as biomarker molecules for the *in situ* detection of this NOB in natural environments. Moreover, different sublineages can be identified by certain combination of these acids. For instance, two cultures in the same sublineage, *N. calida* and GaII (sublineage VI), exhibited considerable differences in their fatty acids profile [25].

Beside *N. marina* and its close relative Ecomares 2.1, the culture S11 from the Laptev Sea is the third culture of the marine *Nitrospira*-sublineage IV, of which a fatty acids profile has been generated. This profile and profiles of the two enrichment cultures from the shrimp biofilter (black and white biocarriers) were similar to the profiles of the other two *Nitrospira* strains originating from marine environments. The specific fatty acids of *Nitrospira* sublineage IV consisted of the two marker lipids 16∶1 *cis*7 and 16∶0 *cis*11 in combination with the non-specific fatty acid 16∶0 as major compounds in the membranes (Table 1). This characteristic pattern (Table 3) is distinguishable from the lipid profiles of the sublineages I, II, V and VI [10], [24]–[25]. The lipid pattern of sublineage III is not yet determined due to the lack of enrichment cultures for this group. The marker lipid analyses confirmed the possibility of differentiation of marine *Nitrospira* strains of sublineage IV from other *Nitrospira* sublineages using this method.

**Table 3:** Marker lipids for *Nitrospira* species from whole fatty acid profiles.

		16∶1 *cis*7	16∶1 *cis*11	16∶0	16∶0 11methyl
sublineage I	*Candidatus* Nitrospira defluvii^a^		**++** e	**+**	
sublineage II	*Nitrospira moscoviensis* b		**++**	**+**	**++**
sublineage IV	*Nitrospira marina* b	**++**	**+**	**++**	
	Enrichment Ecomares 2.1^c^	**++**	**++**	**+**	
	Enrichment S11	**++**	**++**	**+**	
	Enrichment Sh black	**++**	**+**	**++**	
	Enrichment Sh white	**+**	**+**	**++**	
sublineage V	*Candidatus* Nitrospira bockiana^b^	**+**		**++**	**++**
sublingeage VI	*Nitrospira calida* d	**+**		**++**	**++**
	Enrichment Ga II^d^	**++**		**++**	

a data from Spieck *et al.* [10].

b data from Lipski *et al.* [24].

c data from Keuter *et al.* [5] .

d data from Lebedeva *et al.* [25].

e +: marker lipids with percentage ≤20%; ++: marker lipids with >20% of whole fatty acids.

### Conclusion

The labeling approach with ^13^C as substrate indicated the presence and the metabolic activity of a *Nitrospira*-related population as the dominant nitrite-oxidizer in analyzed brackish moving-bed filter systems. The abundance of *Nitrospira*-like organism was shown under various incubation parameters. The sequencing approach of the 16S rRNA genes revealed four *Nitrospira* phylotypes, but only the second cluster was restricted to the specific incubation conditions of low temperature. Our study could also confirm that whole fatty acid profiles of currently known *Nitrospira* sublineage IV organisms from marine habitats always consist of the two marker lipids 16∶1 *cis*7 and 16∶1 *cis*11 combined with the non-specific acid 16∶0 as major compounds in the membranes.

## Supporting Information

Figure S1 **New high density polyethylene biocarries.** Biocarriers of the type HX09KL (Stöhr, Marktrodach, Germany) made of new material (white) and of recycled material (black color, due to the addition of 3% carbon black). Scale of the ruler in centimeter (cm).
(TIF)

Figure S2 **Nitrite oxidizing potentials of black and white biocarriers from the shrimp biofilter.** 10 biocarriers were shaken in 50 ml mineral medium spiked with 1 mM nitrite. Bars right axis: nitrite-oxidizing potentials (in nmol substrate per hour) of NOB on 1 recycled (stripes) or new (dots) HDPE biocarrier. Left axis; nitrate concentrations (black line) of the biofilter water indicating the N load of the system over the sampling period of one year.
(TIF)
