# Supplementary figures and images for: Relevance and Diversity of Nitrospira Populations in Biofilters of Brackish RAS
Source: PLoS One. 2013 May 21;8(5):e64737. doi: 10.1371/journal.pone.0064737 (PMC3660363; doi:10.1371/journal.pone.0064737)

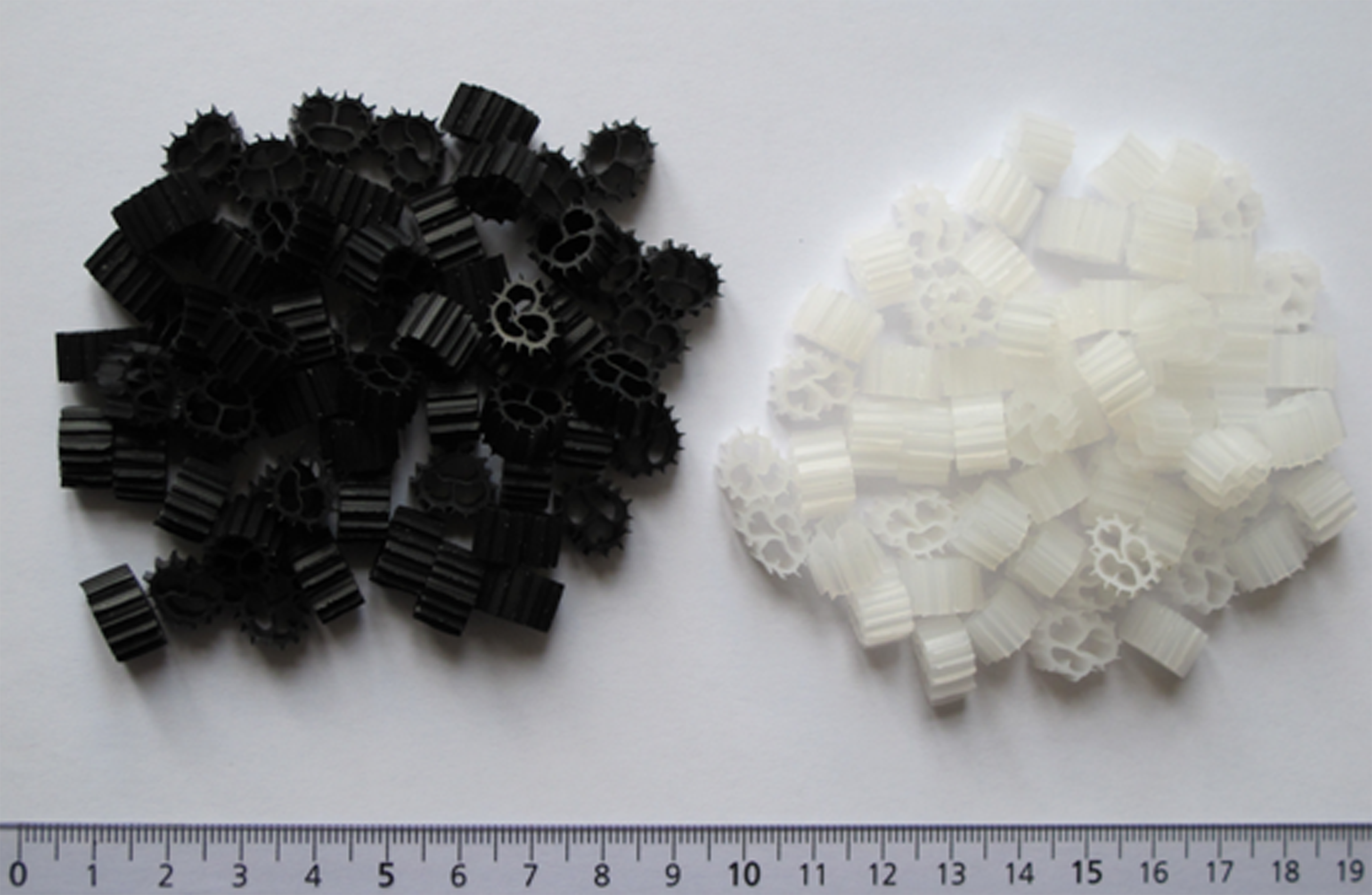

Supplement: Figure S1 — New high density polyethylene biocarries. Biocarriers of the type HX09KL (Stöhr, Marktrodach, Germany) made of new material (white) and of recycled material (black color, due to the addition of 3% carbon black). Scale of the ruler in centimeter (cm). (TIF) [file pone.0064737.s001.tif]

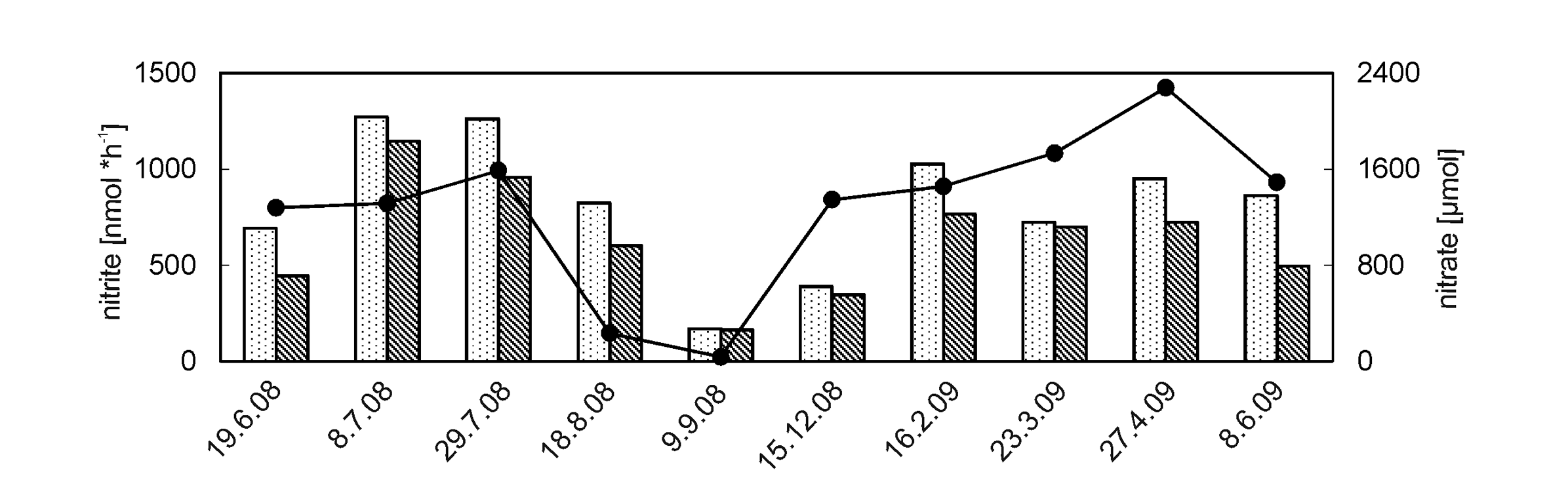

Supplement: Figure S2 — Nitrite oxidizing potentials of black and white biocarriers from the shrimp biofilter. 10 biocarriers were shaken in 50 ml mineral medium spiked with 1 mM nitrite. Bars right axis: nitrite-oxidizing potentials (in nmol substrate per hour) of NOB on 1 recycled (stripes) or new (dots) HDPE biocarrier. Left axis; nitrate concentrations (black line) of the biofilter water indicating the N load of the system over the sampling period of one year. (TIF) [file pone.0064737.s002.tif]
